# Role of succinate in airway epithelial cell regulation following traumatic lung injury

**DOI:** 10.1172/jci.insight.166860

**Published:** 2023-09-22

**Authors:** Madathilparambil V. Suresh, Sinan Aktay, George Yalamanchili, Sumeet Solanki, Dily Thazhath Sathyarajan, Manikanta Swamy Arnipalli, Subramaniam Pennathur, Krishnan Raghavendran

**Affiliations:** 1 Department of Surgery,; 2 Department of Molecular & Integrative Physiology, and; 3 Internal Medicine-Nephrology, University of Michigan, Ann Arbor, Michigan, USA.

**Keywords:** Inflammation, Pulmonology, Hypoxia, Macrophages

## Abstract

Lung contusion and gastric aspiration (LC and GA) are major risk factors for developing acute respiratory distress following trauma. Hypoxia from lung injury is mainly regulated by hypoxia-inducible factor 1α (HIF-1α). Published data from our group indicate that HIF-1α regulation in airway epithelial cells (AEC) drives the acute inflammatory response following LC and GA. Metabolomic profiling and metabolic flux of Type II AEC following LC revealed marked increases in glycolytic and TCA intermediates in vivo and in vitro that were HIF-1α dependent. GLUT-1/4 expression was also increased in HIF-1α^+/+^ mice, suggesting that increased glucose entry may contribute to increased intermediates. Importantly, lactate incubation in vitro on Type II cells did not significantly increase the inflammatory byproduct IL-1β. Contrastingly, succinate had a direct proinflammatory effect on human small AEC by IL-1β generation in vitro. This effect was reversed by dimethylmalonate, suggesting an important role for succinate dehydrogenase in mediating HIF-1α effects. We confirmed the presence of the only known receptor for succinate binding, SUCNR1, on Type II AEC. These results support the hypothesis that succinate drives HIF-1α–mediated airway inflammation following LC. This is the first report to our knowledge of direct proinflammatory activation of succinate in nonimmune cells such as Type II AEC in direct lung injury models.

## Graphical abstract

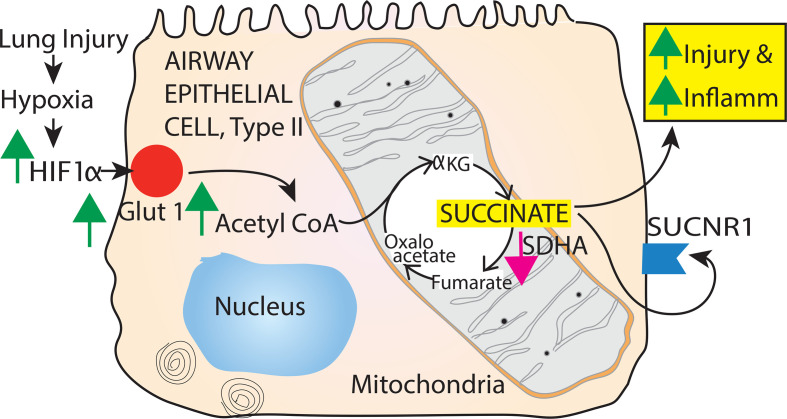

## Introduction

Acute respiratory distress syndrome (ARDS) is characterized by the development of a diffuse, inflammatory lung injury, leading to increased pulmonary vascular permeability and increased lung weight. These effects result in arterial hypoxemia and bilateral radiographic opacities with increased physiological dead space and decreased lung compliance (1). Direct injury to the lung and indirect risk factors (sepsis, pancreatitis) promote the development of ARDS. In a patient with blunt or penetrating trauma, lung contusion and gastric aspiration (LC and GA) are the most common direct etiologic factors for developing ARDS (2, 3). In its most severe form, ARDS has a mortality rate of between 40% and 46% (4). Therefore, understanding the biological basis of ARDS is critical.

Hypoxia-inducible factor 1α (HIF-1α) is a dimeric protein that is the master regulator of numerous hypoxic and inflammatory response genes (5–7). HIF-1α is crucial in regulating the immune response in hypoxic situations, making it particularly important to consider in the context of LC (5–7). We have previously reported the role of HIF-1α in stress-induced hypoxia and the importance of alveolar epithelial cells (AEC) as lung-injury models (8). Furthermore, Type II AEC showed inflammatory upregulation through HIF-1α (9).

In vivo and in vitro studies report that lung cells are relatively tolerant to severe and prolonged hypoxia, with no change in the ultrastructural characteristics, cell viability, or adenosine triphosphate (ATP) content (10). In several cell types, tolerance to hypoxia has been assumed to be the result of the ability of the cell to cope with a decrease in available energy due to the limitation of oxidative phosphorylation by adaptive strategies that maintain ATP supply (11). There are 2 general mechanisms that cells invoke to maintain ATP levels. First, they increase glycolysis by upregulating glycolytic enzymes and glucose transporters, and they increase oxygen supply by increasing vascular endothelial growth factor (VEGF) and angiogenesis. Second, they decrease the activity of some ATP-consuming proteins, such as the Na^+^K^+^-ATPase, thus decreasing cellular respiration and oxygen demand.

LC has previously been found to rapidly induce profound global hypoxia (8). Previous experiments in our lab found that increased expression of HIF-1α from lung samples was observed as early as 60 minutes after the insult (9). The extent of lung injury following LC and release of proinflammatory cytokines was significantly reduced in HIF-1α–conditional KO (HIF-1α–cKO)mice. Global hypoxia was present after LC, but hypoxic foci were not uniform (9). Regarding AEC, hypoxic AEC preferentially undergo apoptosis. The expression of proteins involved in HIF-related pathways and inflammasome activation was significantly increased in hypoxic AEC.

Additionally, aspiration-induced lung injury is a substantial risk factor for ARDS. In our recent study, we studied C57BL/6 mice, hypoxia reporter mice (oxygen-dependent degradation [ODD] domain within HIF-1α [ODD-Luc]), and HIF-1α triple transgenic cKO mice specific in Type II AEC to confirm and characterize the fate of hypoxic AEC in the pathogenesis of acid aspiration–induced lung injury (9). We found that HIF-1α is critical in regulating the inflammatory response following acid aspiration–induced lung injury (9, 12, 13).

Based on these previous findings, we set out to determine the role of Type II AEC in the mitochondrial TCA and glycolysis processes in the presence and absence of HIF-1α following LC and acid aspiration (8, 9, 13). We conclude that hypoxia in mice following direct lung injury promoted the accumulation of succinate. This process was dependent on the activation of HIF-1α in Type II AEC. The process was facilitated by a reduction in succinate dehydrogenase A (SDHA) promoting succinate accumulation. Succinate directly induced inflammation and injury to the surrounding AEC through the presence of succinate receptor 1 (SUCNR1) in AEC, thereby worsening inflammation and permeability injury.

## Results

### Upregulated glycolysis in the presence of HIF-1α following LC.

In our previous studies, we confirmed that doxycycline will induce recombination within the conditional HIF-1α–KO (HIF-1α^–/–^) group. Mice were exposed to doxycycline for 7 weeks starting from P1. The lungs of HIF-1α^+/+^ and HIF-1α^–/–^ mice were then removed and analyzed for HIF-1α expression via IHC. HIF-1α^+/+^ mice showed pronounced HIF-1α expression in the Clara cells and Type II cells of the alveoli. In contrast, the cKO mice showed a marked decrease in expression in these cells and little or no staining in the alveoli. We have previously found that HIF-1α plays a crucial role in mediating the acute inflammatory response following LC and acid aspiration (8). We also found that the downregulation of HIF-1α in Type II cells reduced lung injury and inflammation, suggesting that HIF-1α is a driver of inflammation (8, 9). Therefore, the following experiments aimed to determine the role of HIF-1α in adjusting the metabolism of Type II AEC following LC. HIF-1α^+/+^ and HIF-1α^–/–^ mouse Type II AEC were collected at 0, 24, 48, and 72 hours following LC. Type II AEC extracts were subjected to liquid chromatography with mass spectrometry (MS) analysis of central carbon metabolism.

**Figure 1:**
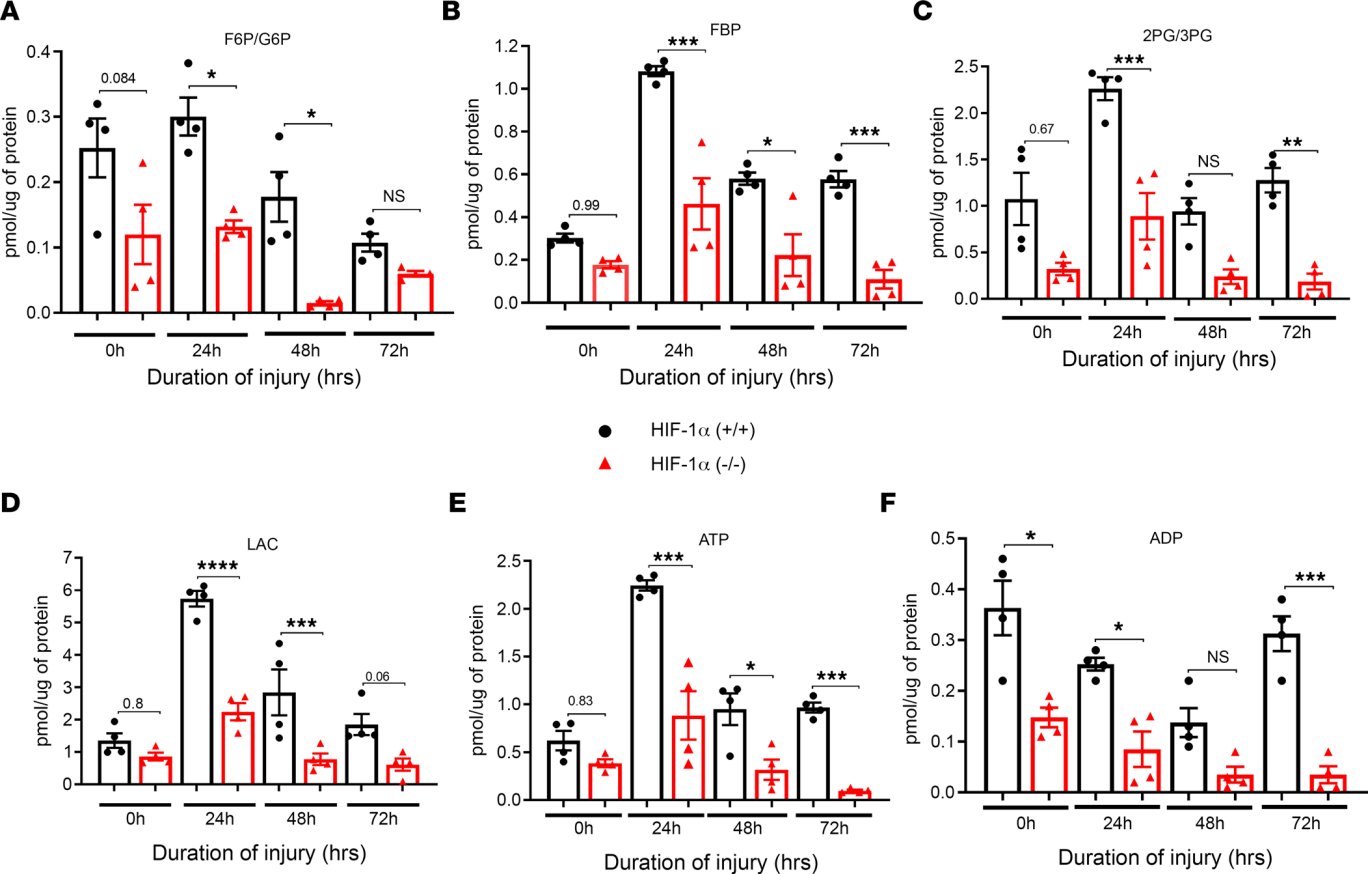
Presence of HIF-1α in AEC regulate anaerobic metabolic profile. HIF-1α^+/+^ and HIF-1α^–/–^ mice were subjected to LC, and Type II AEC cells were collected at 0, 24, 48, and 72 hours (*n* = 4). Type II AEC extracts were subjected to liquid chromatography–MS analysis. (**A**–**F**) Glycolytic and TCA cycle intermediates were analyzed as follows: fructose-6-phosphate/glucose-6-phosphate (F6P/G6P) (**A**), fructose 1,6-bisphosphate (FBP) (**B**), conversion of 3-phosphoglycerate (3PG) to 2PG (**C**), lactate (LAC) (**D**), ATP (**E**), and ADP (**F**). The statistical significance of data was analyzed using 2-way ANOVA with Tukey’s multiple-comparison tests. *P < 0.05, **P < 0.01, ***P < 0.001, and ****P < 0.0001.

Glycolysis is the first phase of cellular respiration that converts glucose into pyruvate. The free energy released in this process forms the high-energy compounds ATP and nicotinamide adenine dinucleotide (NADH). ATP is the fundamental energy currency of cellular activity, and adenosine has been demonstrated to play essential roles in human physiology and pathophysiology (14, 15). We found significantly increased fructose-6-phosphate/glucose-6-phosphate (F6P/G6P) levels in the HIF-1α^+/+^ mice at 24 and 48 hours compared with the HIF-1α^–/–^ mice following LC (Figure 1A). Next, we examined fructose 1,6-bisphosphate (FBP), an endogenous intermediate of the glycolytic pathway. The levels of FBP were significantly higher at 24, 48, and 72 hours following LC in the HIF-1α^+/+^ mice compared with the HIF-1α^–/–^ mice (Figure 1B). Additionally, converting 3-phosphoglycerate (3PG) to 2PG is an essential cellular reaction in the glycolytic pathway. Our data show the levels of 3PG and 2PG were also significantly elevated at the 24- and 72-hour time points following LC in the HIF-1α^+/+^ mice compared with the HIF-1α^–/–^ mice (Figure 1C). Next, we explored the role of lactate, a glycolytic intermediate, in Type II cells following LC. Lactate is one of the most enriched byproducts of cellular metabolism in inflamed tissues. Its accumulation leads to the exacerbation of the inflammatory response in chronic inflammatory disorders (16). Levels of lactate were significantly higher in the HIF-1α^+/+^ mice at the 24- and 48-hour time points following LC when compared with the HIF-1α^–/–^ mice (Figure 1D).

HIF-1α increases mitochondrial metabolism in LC. Previous studies show that inflammatory and hypoxic conditions lead to increased release of ATP/ADP (9, 10). Here, we examined the role of purinergic signaling in acute pulmonary inflammation following LC. Our data show that the levels of ATP and adenosine diphosphate (ADP) were higher in the HIF-1α^+/+^ mice at all the time points except for 0 hours for ATP and 48 hours for ADP compared with the HIF-1α^–/–^ mice (Figure 1, E and F).

**Figure 2:**
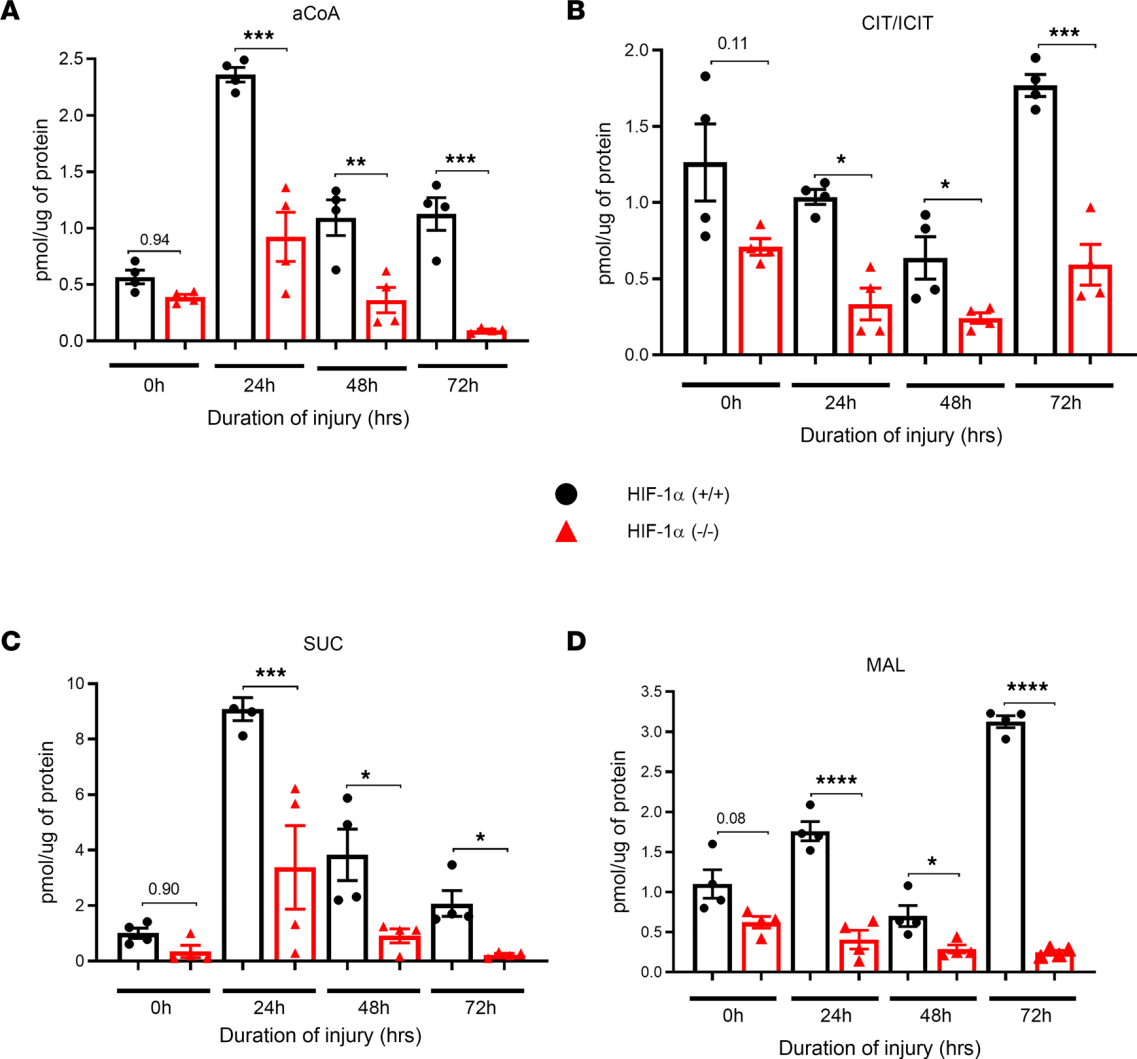
Presence of HIF-1α in AEC regulate anaerobic metabolic profile. HIF-1α^+/+^ and HIF-1α^–/–^ mice were subjected to LC, and Type II AEC were collected (*n* = 4). Type II AEC extracts were subjected to liquid chromatography–MS analysis. (**A**–**D**) The following TCA cycle intermediates were analyzed: acetyl-CoA (aCoA) (**A**), citrate and isocitrate (CIT/ICIT) (**B**), succinate (SUC) (**C**), and malate (MAL) (**D**). The statistical significance of data was analyzed using 2-way ANOVA with Tukey’s multiple-comparison tests. *P < 0.05, **P < 0.01, ***P < 0.001, and ****P < 0.0001.

The TCA cycle is a metabolic hub in the mitochondrial matrix that helps generate ATP as an end product of oxidative phosphorylation. Citrate is a critical metabolite to support mitochondrial bioenergetics and cytosolic macromolecular synthesis (17). Acetyl coenzyme A (acetyl-CoA) is a component of cellular respiration that adds acetyl groups to biochemical reactions. These reactions are used to metabolize proteins, carbohydrates, and lipids that will provide energy sources in the forms of ATP, lactic acid, and ketone bodies. We observed significant elevations across many TCA cycle intermediates, including acetyl-CoA, in HIF-1α^+/+^ mice at 24, 48, and 72 hours compared with the corresponding control following LC (Figure 2A). Citrate and isocitrate are intermediates in the TCA. The mitochondrial export of citrate and isocitrate through the citrate-isocitrate carrier (CIC) has been suggested to initiate a key pathway that amplifies glucose-stimulated insulin secretion. Citrate and isocitrate levels were significantly elevated in the HIF-1a^+/+^ mice at 24, 48, and 72 hours compared with the HIF-1a^–/–^ mice following LC (Figure 2B).

Succinate is an essential metabolite in both host and microbial processes. Although generally regarded as an intermediate, succinate accumulates in certain pathophysiological situations, such as inflammation and metabolic stress (18). We examined the role of succinate in the TCA in Type II AEC cells following LC. Our data show that the level of succinate was significantly greater in the HIF-1α^+/+^ mice at 24, 48, and 72 hours compared with the HIF-1α^–/–^ mice following LC (Figure 2C). As an intermediate of the TCA cycle, malate is intimately associated with mitochondrial energy metabolism and is also the origin of carbon skeletons exported from the mitochondrion in support of amino acid biosynthesis (19). Malate levels were significantly higher in the HIF-1α^+/+^ mice at the 24-, 48-, and 72-hour time points following LC when compared with the HIF-1α^–/–^ mice (Figure 2D).

### HIF-1α activates the pentose phosphate pathway following LC.

**Figure 3:**
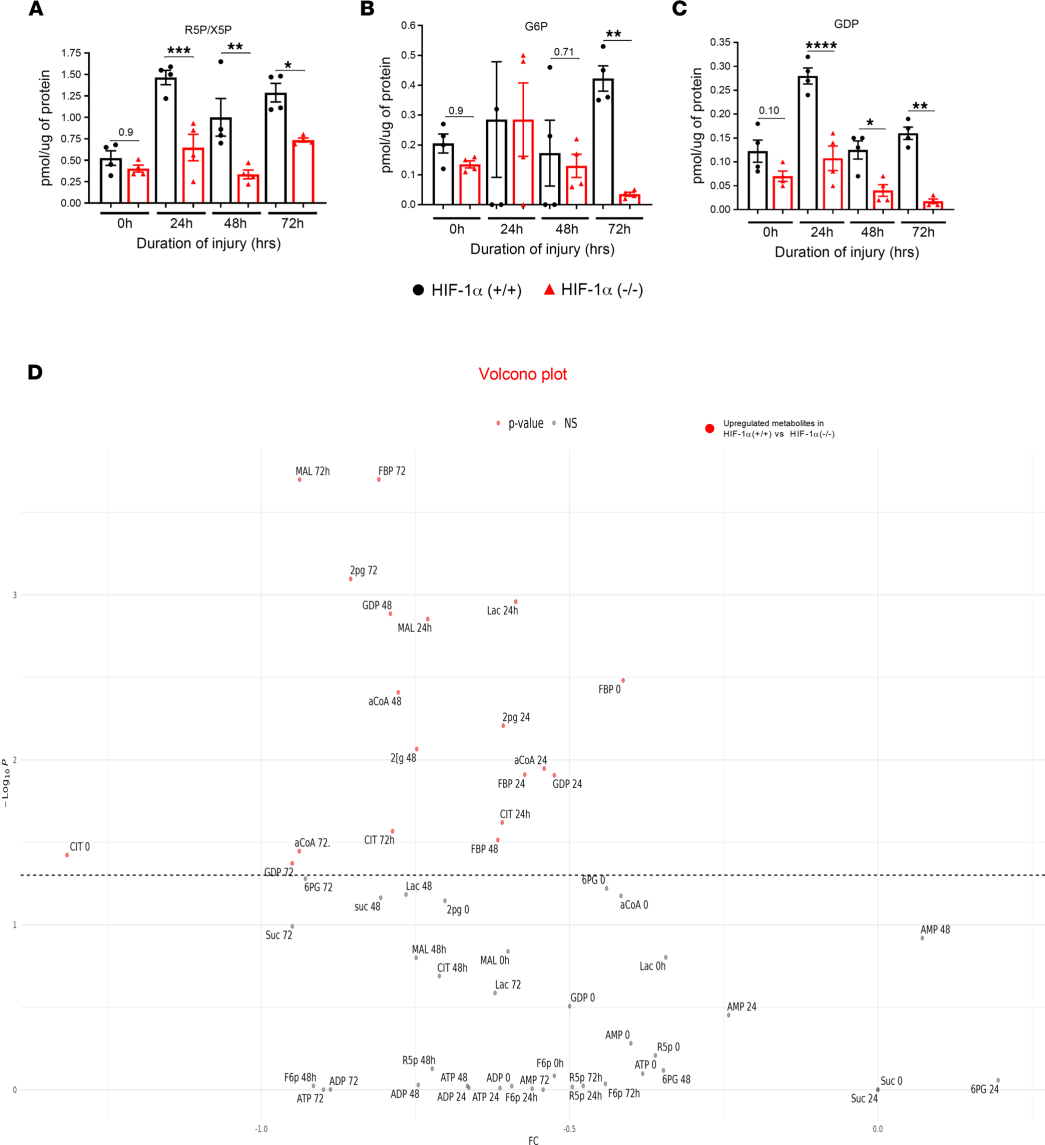
HIF-1α on activating the pentose phosphate pathway following LC. HIF-1α^+/+^ and HIF-1α^–/–^ mice were subjected to LC, and Type II AEC were collected (*n* = 4). Type II AEC extracts were subjected to liquid chromatography–MS analysis. (**A**–**C**) The pentose phosphate pathway intermediates were analyzed as follows: ribose 5-phosphate (R5P/X5P) (**A**), glucose 6-phosphate (G6P) (**B**), and guanosine diphosphate (GDP) (**C**). (**D**) The volcano plot shows the difference in metabolite levels between the HIF-1α^+/+^ and HIF-1α^–/–^ groups at a specific time organized by *P* value. All metabolites were higher in the HIF-1α^+/+^ group than in the HIF-1α^–/–^ group. HIF-1α^+/+^ and HIF-1α^–/–^ mice were subjected to LC and Type II AEC collected at 24 hours (*n* = 4). *P < 0.05, **P < 0.01, ***P < 0.001, and ****P < 0.0001. The statistical significance of data was analyzed using 2-way ANOVA with Tukey’s multiple-comparison tests.

The pentose phosphate pathway (PPP), also known as the pentose phosphate shunt, is primarily considered an anabolic pathway for producing nucleotides and aromatic amino acids (20). 6-Phosphogluconate is an intermediate in the PPP (21). Under normal cell metabolism, 6-phosphogluconate entering the oxidative branch of the PPP is converted to ribose 5-phosphate (R5P) and xylulose 5-phosphate (X5P). We examined the role of R5P/X5P in Type II cells following LC. Here, we found that the R5P levels were significantly higher at 24, 48, and 72 hours in the HIF-1α^+/+^ mice compared with the HIF-1α^–/–^ mice following LC (Figure 3A). The PPP branches after the first step of glycolysis and consumes the intermediate glucose 6-phosphate (G6P) to generate fructose 6-phosphate (F6P) and glyceraldehyde 3-phosphate (G3P) through the oxidative and nonoxidative branches of the PPP (20). We examined the level of the G6P following LC. Our data indicate that there was a significantly higher G6P level only at the 72-hour time point in the HIF-1α^+/+^ mice following LC compared with the HIF-1α^–/–^ mice (Figure 3B).

Additionally, guanosine diphosphate (GDP) and guanosine triphosphate (GTP) metabolites play a vital role in many biological functions. GTP is essential for signal transduction, particularly with G proteins, where it acts in second-messenger mechanisms and is converted to GDP through GTPases. The data show that the GDP level was significantly higher in the HIF-1α^+/+^ mice at 24, 48, and 72 hours following LC than in the HIF-1α cKO mice (Figure 3C). We also present the metabolomic data using a volcano plot, which is a scatter plot that displays the significance (*P* value) and the amount of change (fold change). The plot shows the difference in metabolite levels between the HIF-1α^+/+^ and HIF-1α^–/–^ groups at a specific time. All metabolites were higher in the HIF-1α^+/+^ group than in the HIF-1α^–/–^ group (Figure 3D). Overall, these results demonstrate that, with the deletion of HIF-1α in Type II AEC, there is a marked anabolic predisposition toward the generation of nucleotides and aromatic amino acids.

### HIF-1α^–/–^ mice show a reduction in glucose transporter gene expression following LC.

**Figure 4:**
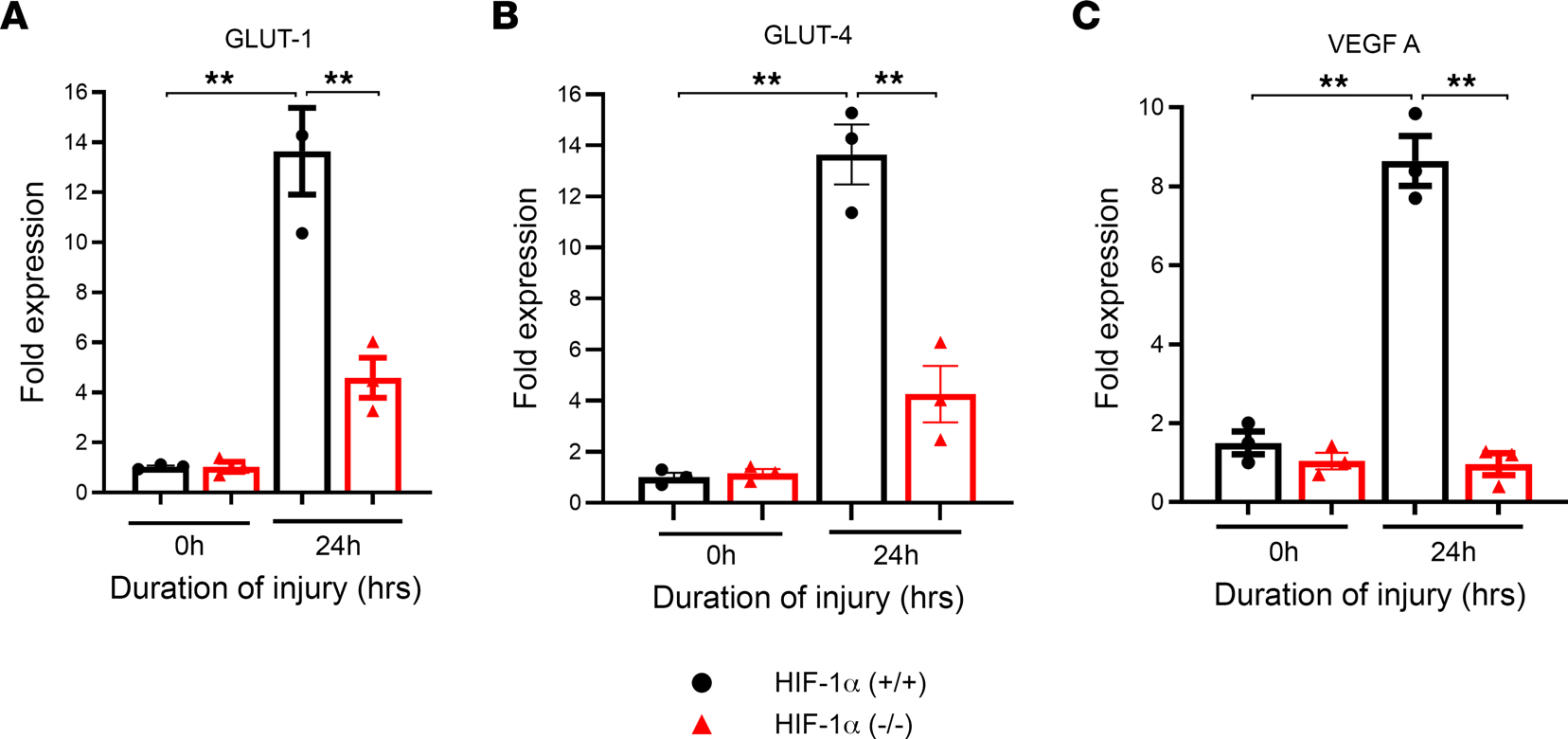
HIF-1α deletion shows a reduction in glucose transporter gene level following LC. The isolated type 2 cells from HIF-1α^+/+^ and HIF-1α^–/–^ mice were measured for gene expression following LC. (**A**–**C**) The expression levels of GLUT-1 (**A**), GLUT-4 (**B**), and VEGFA (**C**) were determined by qPCR (*n* = 3). The statistical significance of data was analyzed using 2-way ANOVA with Tukey’s multiple-comparison tests. **P < 0.01.

Given that increased cellular glucose entry may activate downstream glycolysis, mitochondrial oxidative phosphorylation, and the PPP, we went on to examine whether HIF-1α facilitates intracellular glucose entry. Glucose is primarily transported across the plasma membrane by facilitative glucose transporters (22). We investigated the role of glucose transporter 1 and 4 (GLUT-1/4) and VEGFA in the glucose metabolism of Type II AEC following LC. HIF-1α^+/+^ and HIF-1α^–/–^ mice had RNA isolated from their Type II AEC following LC. A quantitative reverse transcriptase PCR (RT-PCR) measured GLUT-1, GLUT-4, and VEGFA levels. We found that the expression of GLUT-1 and GLUT-4 was significantly reduced at the 0- and 24-hour time points in the HIF-1α^–/–^ mice compared with the HIF-1α^+/+^ mice following LC in Type II AEC (Figure 4, A and B). Previous studies show that VEGFA is highly expressed in tumors and enhances glycolysis in cancer cells with HIF-1α upregulation. Here we found that VEGFA was significantly elevated at all the time points in HIF-1α^+/+^ mice following LC compared with the HIF-1α^–/–^ mice (Figure 4C). These data suggest that, in a hypoxic environment, the expression of specific glucose transporter genes and VEGF increases through the upregulation of HIF-1α, contributing to increased central carbon metabolism.

### HIF-1α increases ^13^C_6_-glucose flux in Type II AEC following LC.

**Figure 5:**
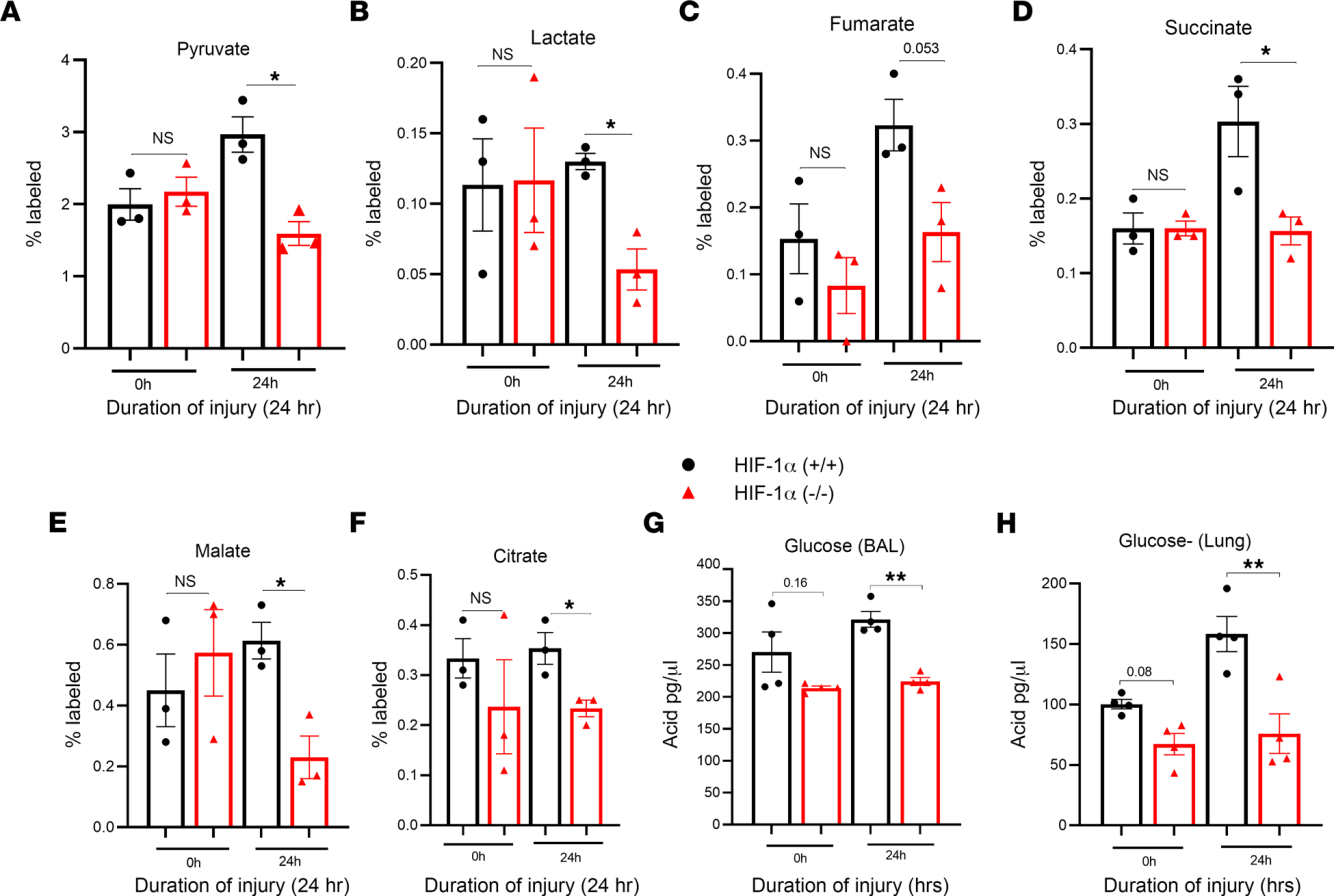
HIF-1α increases ^13^C_6_-glucose flux in Type II AEC following LC. After LC, we conducted measurements on the levels of glycolytic and TCA cycle intermediates in the Type II AEC of both HIF-1α^+/+^ and HIF-1α^–/–^ mice (*n* = 3). (**A**–**F**) We analyzed the intermediates and found pyruvate (**A**), lactate (**B**), fumarate (**C**), succinate (**D**), malate (**E**), and citrate (**F**). During the experiment, acid was given to both the HIF-1α^+/+^ and HIF-1α^–/–^ mouse groups (*n* = 4). (**G** and **H**) Samples from their bronchoalveolar lavage (BAL) and lung lysate were collected and analyzed to determine their glucose levels. After analyzing the data, we used the unpaired *t* test with Welch’s correction to determine its statistical significance. *P < 0.05 and **P < 0.01.

Next, we conducted experiments using ^13^C_6_ isotope–based metabolic flux analysis to understand how glucose is metabolized in Type II AEC after LC. To facilitate this, we added 5 mM of ^13^C_6_-glucose to the Type II epithelial cell culture medium, allowing the cells to metabolize it for an hour. We then quantified the relative abundances of isotopologues from ^13^C_6_-glucose catabolism and analyzed glycolytic and TCA cycle metabolites using a quadrupole TOF mass spectrometer. Type II AEC derived from HIF-1α^+/+^ mice had a greater amount of labeling for glycolytic metabolites at the 24-hour time point compared with the HIF-1α^–/–^ mice after LC (Figure 5, A and B). Next, we measured the levels of TCA cycle intermediates such as fumarate, succinate, malate, and citrate. The Type II AEC of HIF-1α^+/+^ mice displayed increased incorporation of the ^13^C_6_ label into TCA cycle metabolites after LC in contrast to HIF-1α^–/–^ mice (Figure 5, C–F). These results reinforce the findings of static metabolomic analyses and strongly suggest that, in Type II AEC, the presence of HIF-1α^+/+^ results in a substantial increase in glycolytic and TCA cycle glucose flux when compared with HIF-1α^–/–^ in LC.

### HIF-1α^–/–^ mice show a reduction in glucose level following GA.

We also measured the glucose level of lung samples from HIF-1α mice after acid aspiration, both with and without lung injury. The glucose level was measured using the calorimetric method at 24-hour intervals. Glucose levels were significantly higher in bronchoalveolar lavage (BAL) and the lungs in the HIF-1α^+/+^ mice compared with HIF-1α^–/–^ mice at the 24-hour time point (Figure 5, G and H).

### HSAEC show an increase in the expression of hypoxic genes after exposure to hypoxia.

**Figure 6:**
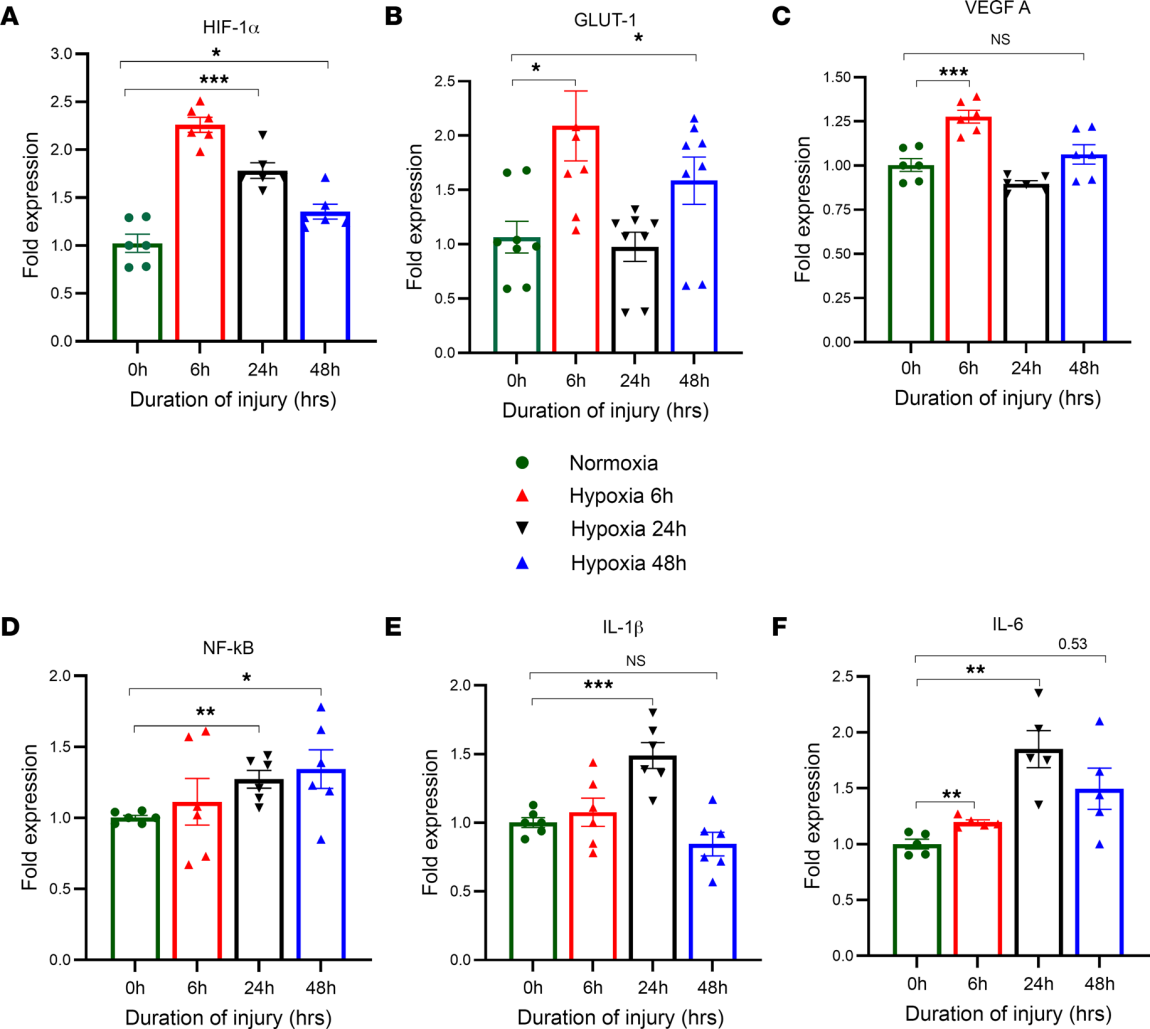
Effect of hypoxia on human small airway epithelial cells. Normal human airway epithelial cells were subjected to hypoxia for 6, 24, and 48 hours. Cell lysates were collected and analyzed for the expression of various hypoxic and inflammatory markers by qPCR (*n* = 6). (**A**–**F**) The expression levels of HIF-1α (**A**), GLUT-1 (**B**), VEGFA (**C**), NF-κB (**D**), IL-1β (**E**), and IL-6 (**F**) were measured. After analyzing the data, we used the unpaired *t* test with Welch’s correction to determine its statistical significance. *P < 0.05, **P < 0.01, ***P < 0.001.

In this experiment, we describe the role of the hypoxic markers following both normoxic and hypoxic conditions in normal human small airway epithelial cells (HSAEC). RNA was isolated from the cell lysate to confirm the fate of hypoxic genes at different time points (6, 24, and 48 hours) following normoxic and hypoxic conditions. The expression of the hypoxic gene HIF-1α was significantly higher at 6, 24 and 48 hours following hypoxia compared with normoxia (Figure 6A). Next, we examined the gene expression level of GLUT-1 proteins responsible for the facilitated diffusion of glucose across a membrane. GLUT-1 gene expression was significantly higher in hypoxic cells at the 6- and 48-hour time points than in the normoxic cells (Figure 6B). We found that VEGFA gene expression was significantly elevated at 6 hours after hypoxia compared with normoxia (Figure 6C).

### HSAEC shows higher levels of inflammatory gene expression after hypoxia.

Additionally, we measured proinflammatory genes such as NF-κB, IL-1β, and IL-6 following hypoxia. NF-κB expression increased dramatically at 24 and 48 hours after hypoxia compared with normoxia (Figure 6D). The level of IL-1β expression was significantly higher at the 24-hour time point following hypoxia compared with normoxia (Figure 6E). The levels of IL-6 expression were also significantly elevated at 6 and 24 hours following hypoxia compared with normoxia (Figure 6F). These data suggest that hypoxia-induced cells show higher cell injury and inflammation than normoxic cells, indicating that HIF-1α is a crucial regulator of hypoxia-inducible genes.

### Increased expression of HIF-1α, GLUT-1, and VEGFA following hypoxia in human airway epithelial cells (A549).

**Figure 7:**
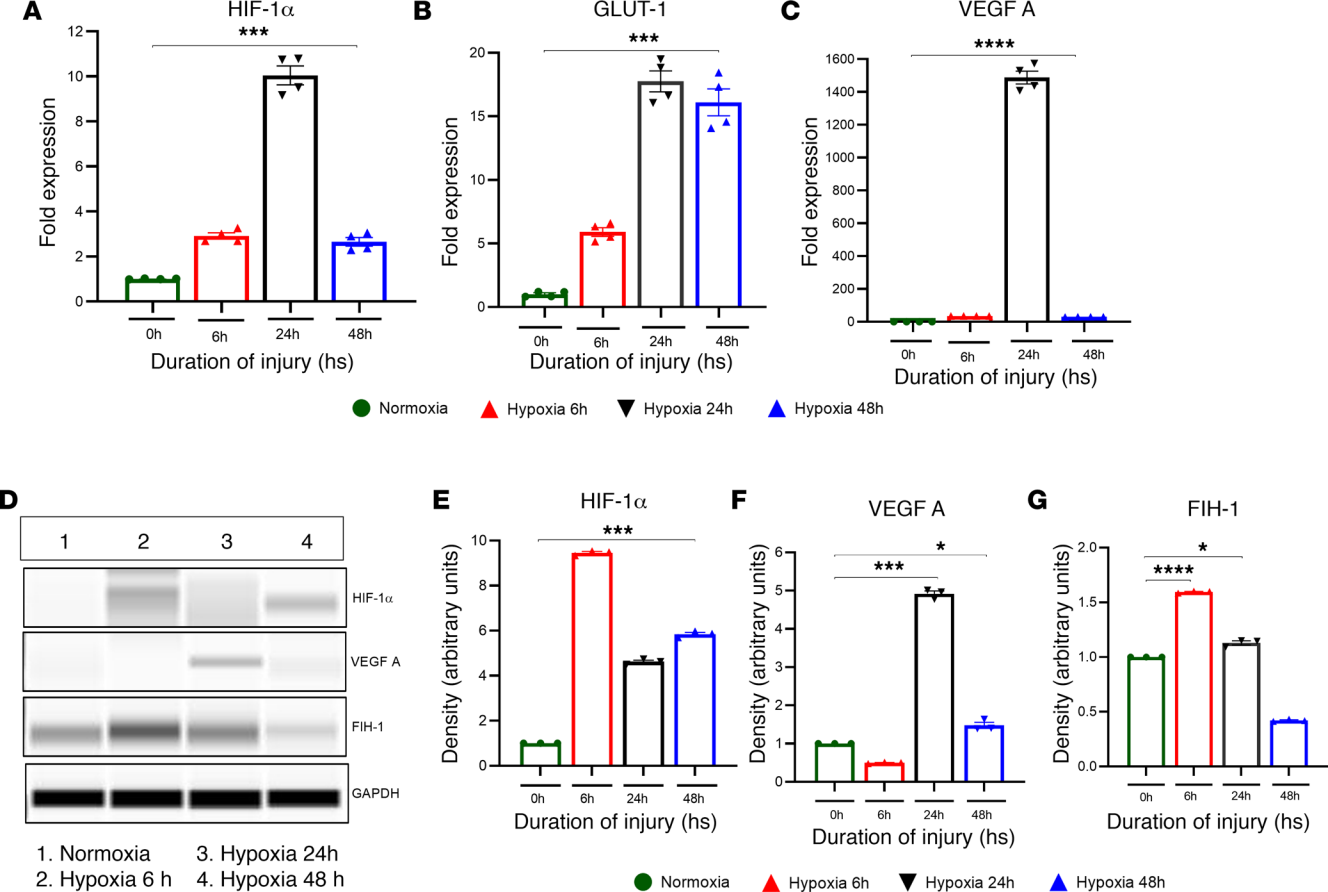
Effect of hypoxia on human airway epithelial cells (A549). A549 cells were subjected to hypoxia for 6, 24, and 48 hours. Cell lysates were collected and analyzed for the expression levels of various hypoxic markers by qPCR (*n* = 4). (**A**–**C**) The expression levels of HIF-1α (**A**), GLUT-1 (**B**), and VEGFA (**C**) were measured. (**D**–**G**) Additionally, whole-lung lysate was used for Western blots to determine the protein expression of HIF-1α, VEGFA, and FIH-1. After analyzing the data, we used the unpaired *t* test with Welch’s correction to determine its statistical significance. *P < 0.05, ***P < 0.001, ****P < 0.0001.

Next, we examined the role of the hypoxic gene in A549. In this study, we determined the role of HIF-1α, GLUT-1, and VEGFA following normoxic and hypoxic conditions. RNA was isolated from the cell lysate to confirm the fate of hypoxic genes at different time points following hypoxic and normoxic conditions. The expression of hypoxic genes such as HIF-1α, GLUT-1, and VEGFA was significantly increased at the 6-, 24-, and 48-hour time points following hypoxia compared with normoxia (Figure 7, A–C).

In an additional experiment, human airway epithelial cells (A549) were subjected to hypoxia. Protein was isolated from whole cells, and a capillary Western blot was conducted to measure the expression of HIF-1α, VEGFA, and factor-inhibiting HIF-1 (FIH-1) (Figure 7D). The expression of HIF-1α was significantly elevated at 6, 24, and 48 hours compared with normoxia (Figure 7E). VEGFA expression was increased only at the 24- and 48-hour time point compared with the control (Figure 7F). FIH-1 expression was significantly increased at the 6- and 24-hour time points in hypoxic conditions compared with the normoxic cells (Figure 7G).

### Succinate directly enhances the expression of the inflammatory gene IL-1β.

**Figure 8:**
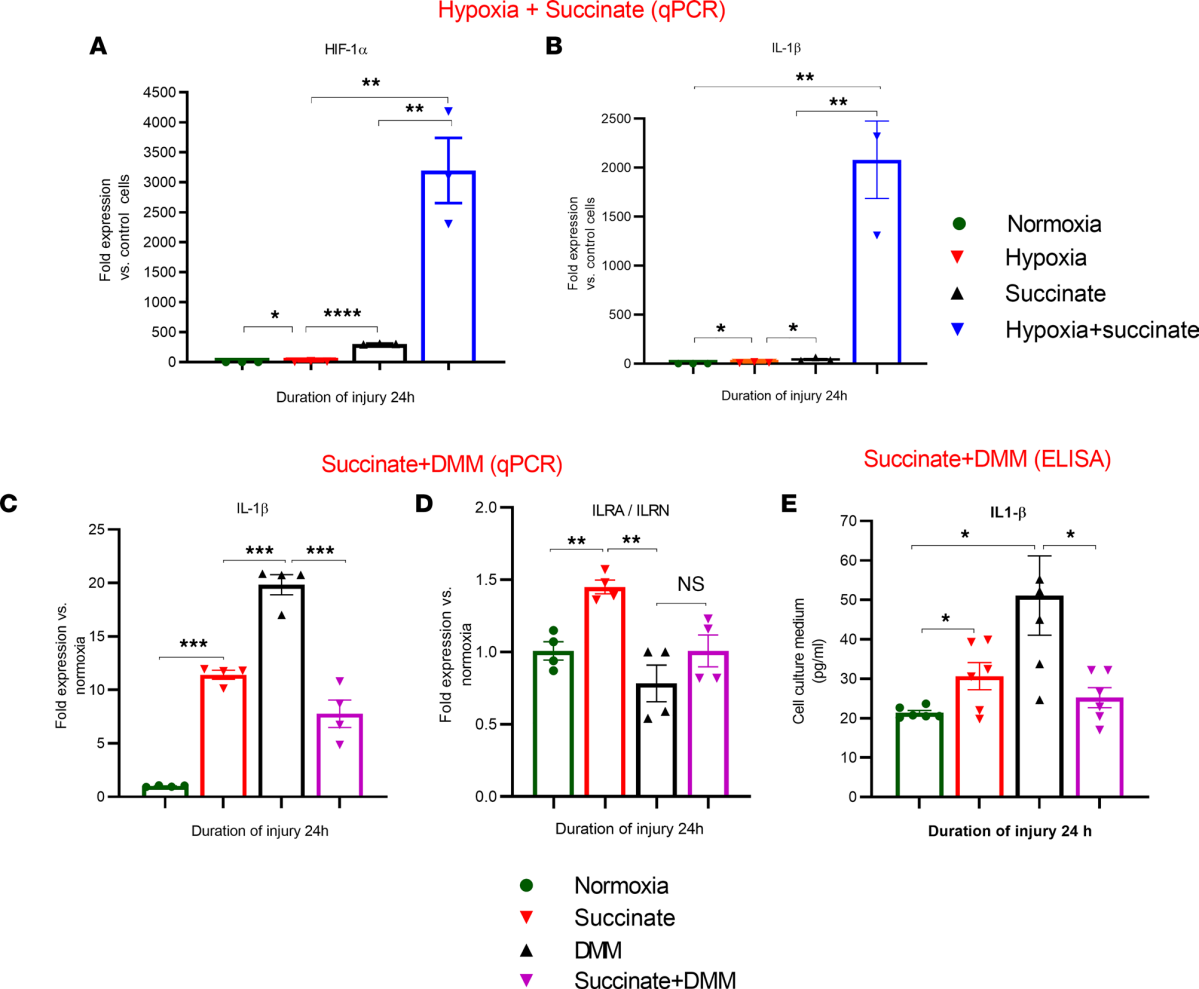
Effect of hypoxia and diethyl succinate on human small airway epithelial cells. Human small airway epithelial cells (HSAEC) were subjected to hypoxia and diethyl succinate (5 mM) for 24 hours (*n* = 3). (**A** and **B**) In the first set of experiments, cells were treated with succinate alone and succinate + hypoxia, and cell lysate was analyzed for IL-1β and HIF-1α by qPCR. (**C** and **D**) In the second set of experiments, cells were treated with dimethyl malonate (DMM) (10 mM), succinate, or succinate + DMM (*n* = 3). The cell lysate was analyzed for IL-1β and ILRA by qPCR. Separately, HSAEC were subjected to treatment with succinate, DMM, and succinate + DMM (*n* = 3). (**E**) Following this step, the medium from the cell culture (*n* = 6) was collected and analyzed for IL-1β using ELISA. After analyzing the data, we used the unpaired *t* test with Welch’s correction to determine its statistical significance. *P < 0.05, **P < 0.01, ***P < 0.001, and ****P < 0.0001.

Diethyl succinate is a neutral molecule used at physiological pH that crosses biological membranes (22). There is a considerable amount of biochemical research on diethyl succinate illustrating the compound’s ability to be incorporated into cells in tissue culture and metabolized by the TCA cycle (17, 23). We performed an experiment on human airway epithelial cells and studied the effect of hypoxia and diethyl succinate on the generation of HIF-1α and IL-1β. The hypoxia + diethyl succinate and diethyl succinate–alone groups showed significantly increased HIF-1α and IL-1β levels compared with the hypoxia group (Figure 8, A and B). In combination with in vivo data in triple transgenic HIF-1α mice, these data strongly indicate that succinate plays a direct role in the AEC regulation of inflammation and injury (Figure 2C).

We studied the effects of hypoxia and succinate treatment on the gene expression of IL-1β and IL-1 receptor antagonist protein (IL-1RN) We conducted the study on A549 cells and exposed them to hypoxia in the presence of succinate (5 mM), diethyl malonate (DMM) (10 mM), and a combination of DMM and succinate. When used alone, hypoxia, succinate, and DMM significantly increased the activation of IL-1β. However, the combination of DMM and succinate resulted in a considerable decrease in the expression of IL-1β compared with the groups treated with DMM and succinate separately. Additionally, our research revealed that the group treated with succinate-only displayed the highest levels of IL-1 receptor antagonist (IL-1RA)/IL-1RN expression. Conversely, the groups treated with DMM and the combination of succinate and DMM showed decreased levels of IL-1RN expression compared with the succinate-only group (Figure 8, C and D).

Finally, we measured the level of IL-1β in the cell culture medium after subjecting A549 cells to succinate, DMM, and a combination of DMM and succinate in separate experiments. After 24 hours, we collected the cell culture medium and measured the expression of IL-1β with ELISA. We found that the levels of IL-1β were higher in the treated groups than in the normoxia group. However, the succinate + DMM treatment reduced the expression of IL-1β compared with cells treated with DMM alone (Figure 8E). Based on the data, using dimethyl malonate as a competitive inhibitor for SDH can reduce the expression of IL-1β at the gene and protein levels in AEC after succinate treatment.

### With hypoxic activation of HIF-1α, there is direct SUCNR1 activation in AEC.

SUCNR1 (also known as GPR91) is a GPCR closely related to the family of P2Y purinoreceptors. SUCNR1 plays a major role as an initiator of transactivation signals releasing extracellular factors and hormones.

**Figure 9:**
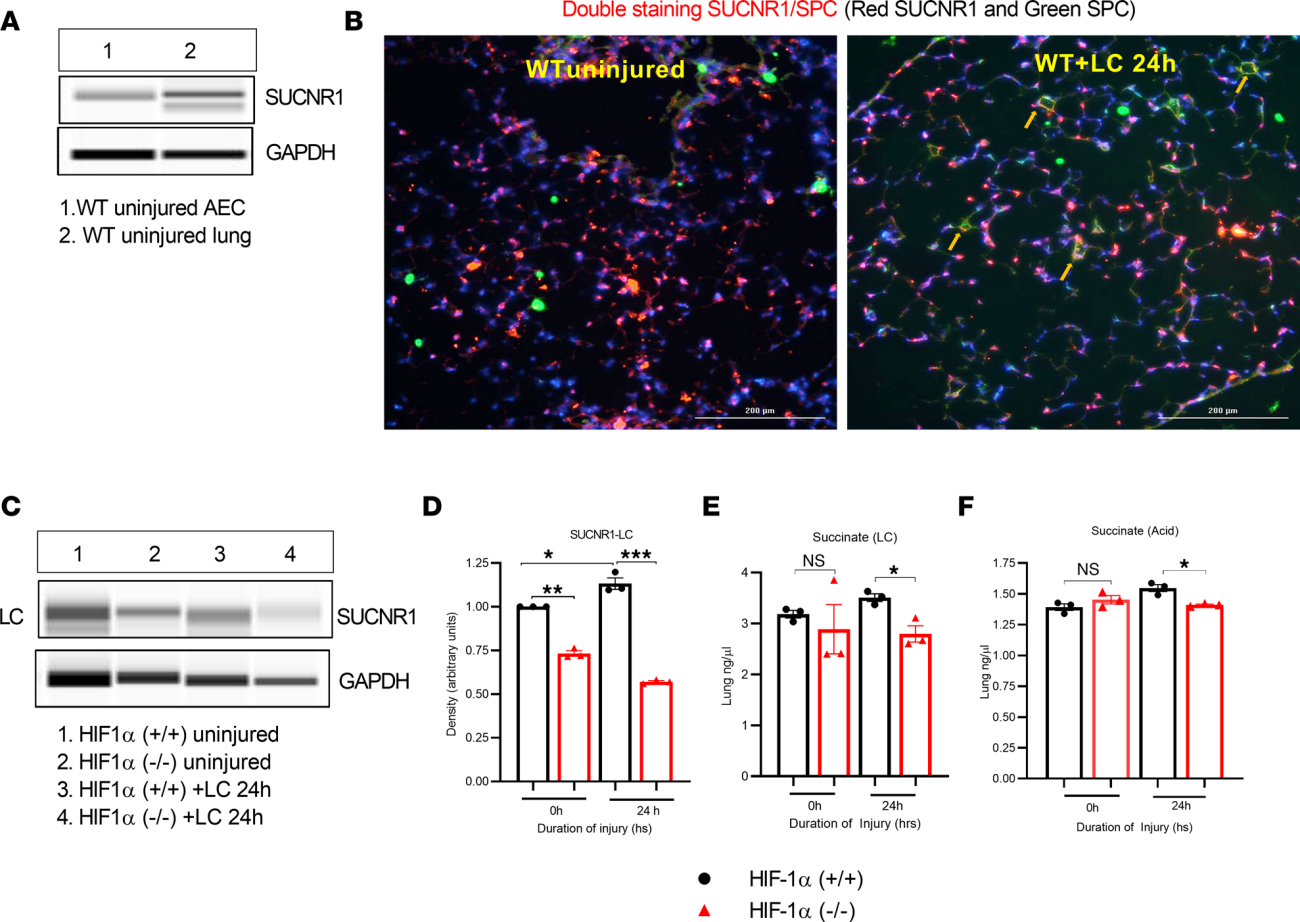
Expression of SUCNR1 receptor in both whole-lung and isolated Type II AEC. (**A**) The capillary Western blot data show that SUCNR1 was detectable in Type II AEC and the entire lung of uninjured C57BL/6 mice. (**B**) After LC, SUCNR1 immunofluorescence staining was performed on a section of a C57BL/6 lung (*n* = 3). Scale bar: 200 µm. (**C** and **D**) Protein expression of SUCNR1 was determined by conducting capillary Western blots on lungs from HIF-1α^+/+^ and HIF-1α^–/–^ mice after LC. (**E** and **F**) Next, the lungs (*n* = 3) from HIF-1α^+/+^ and HIF-1α^–/–^ mice after LC or acid aspiration were measured for succinate expression using a calorimetric assay. After analyzing the data, we used the unpaired *t* test with Welch’s correction to determine its statistical significance. *P < 0.05, **P < 0.01, and ***P < 0.001.

First, we conducted a Western blot analysis to measure the expression of SUCNR1 in the lungs and Type II cells of uninjured WT (C57BL/6) mice. Our results show that there was a substantial level of SUCNR1 expression in both Type II AEC and whole-lung extracts (Figure 9A). Additionally, for 24 hours following LC and uninjured WT mice, we conducted immunostaining to analyze the expression of surfactant protein C (SPC) and SUCNR1 (double staining) in the lungs. Our findings indicate that there was increased expression of SUCNR1 in mice that underwent LC for 24 hours compared with uninjured mice (Figure 9B). We further conducted a study on the levels of SUCNR1 expression in HIF-1α^+/+^ and HIF-1α^–/–^ mice after LC using Western blot analysis. According to the data, HIF-1α^+/+^ mice show significantly higher levels of SUCNR1 expression compared with HIF-1α^–/–^ mice (Figure 9, C and D). In addition, we conducted a calorimetric assay to measure the level of succinate in whole-lung samples taken from HIF-1α^+/+^ and HIF-1α^–/–^ mice after LC or acid aspiration. The data show that the amount of succinate was significantly higher at 24 hours in HIF-1α^+/+^ mice compared with HIF-1α^–/–^ mice after receiving LC and GA (Figure 9, E and F).

### HIF-1α–induced reduction in SDHA contributes to succinate accumulation in direct lung injury.

**Figure 10:**
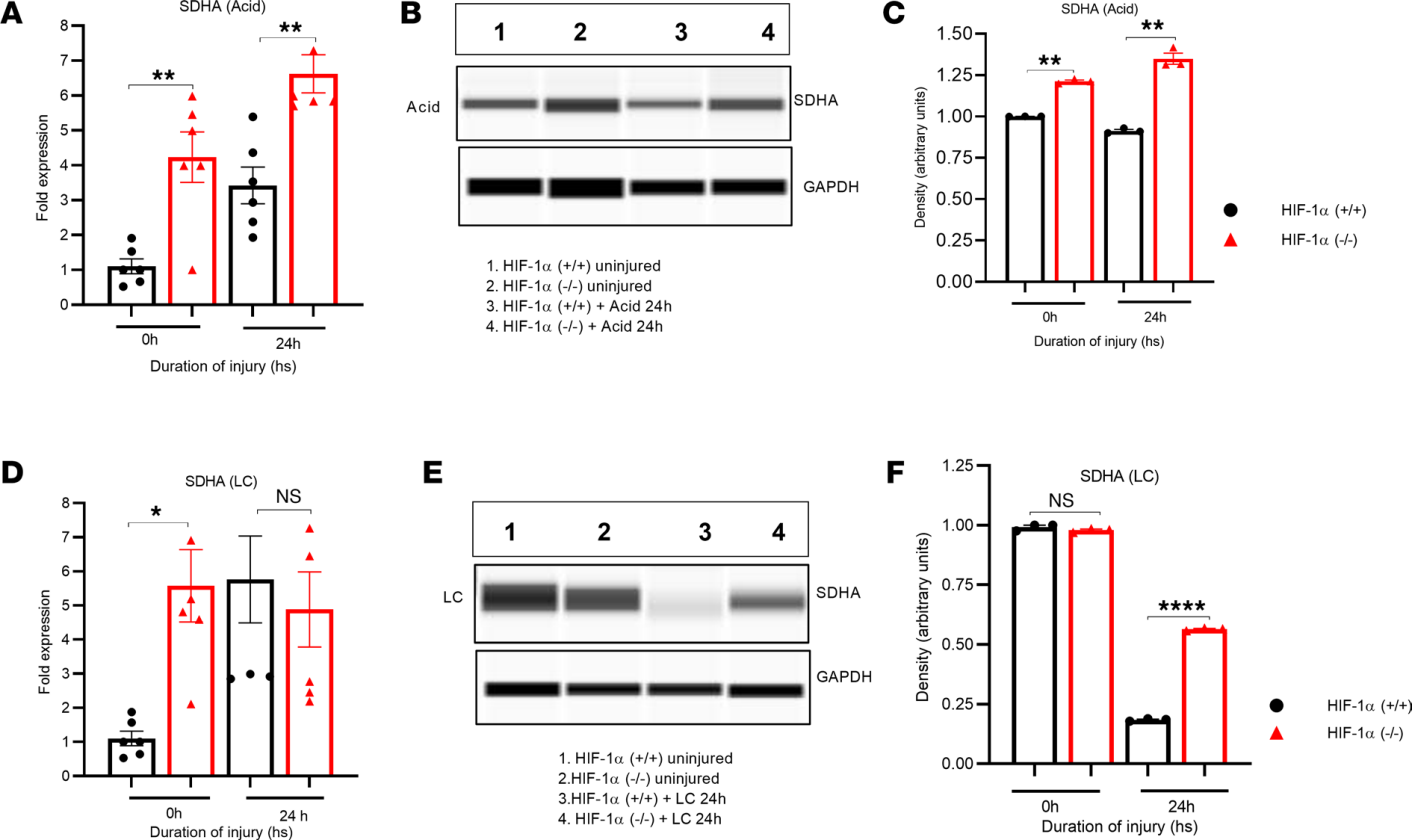
SDHA activity increased when HIF-1α was deleted in response to LC and GA. HIF-1α^+/+^ and HIF-1α^–/–^ mice were subjected to LC or GA. The lungs were digested, and the lysate and RNA were used to determine SDHA (*n* = 6). (**A**–**C**) Following GA, the expression of the SDHA gene was detected through qPCR (**A**), and the protein expression was determined using Western blots (**B** and **C**). (**D**–**F**) After LC, SDHA gene expression was measured via qPCR (**D**), and protein expression was assessed using capillary Western blots (**E** and **F**). *P < 0.05, **P < 0.01, and ****P < 0.0001. After analyzing the data, we used the unpaired *t* test with Welch’s correction to determine its statistical significance.

Metabolic processing of succinate is partly mediated by the enzyme SDH (22). SDH is a component of respiratory complex II found in bacterial cells and the inner mitochondrial membrane of eukaryotes (22). SDH plays an important role in the TCA and respiratory electron transfer chain. Following LC or acid aspiration (along with uninjured controls) in HIF-1α^+/+^ and HIF-1α^–/–^ mice, we analyzed whole-lung samples for gene and protein expression of the 2 most common subunits of SDH: SDHA and SDHB. We found that SDHA gene and protein expression significantly increased in HIF-1α^–/–^ mice after acid aspiration compared with HIF-1α^+/+^ mice (Figure 10, A–C). We also observed higher SDHA protein expression at 24 hours and higher gene expression at 0 hours in HIF-1α^–/–^ mice after LC compared with HIF-1α^+/+^ mice (Figure 10, D–F).

**Figure 11:**
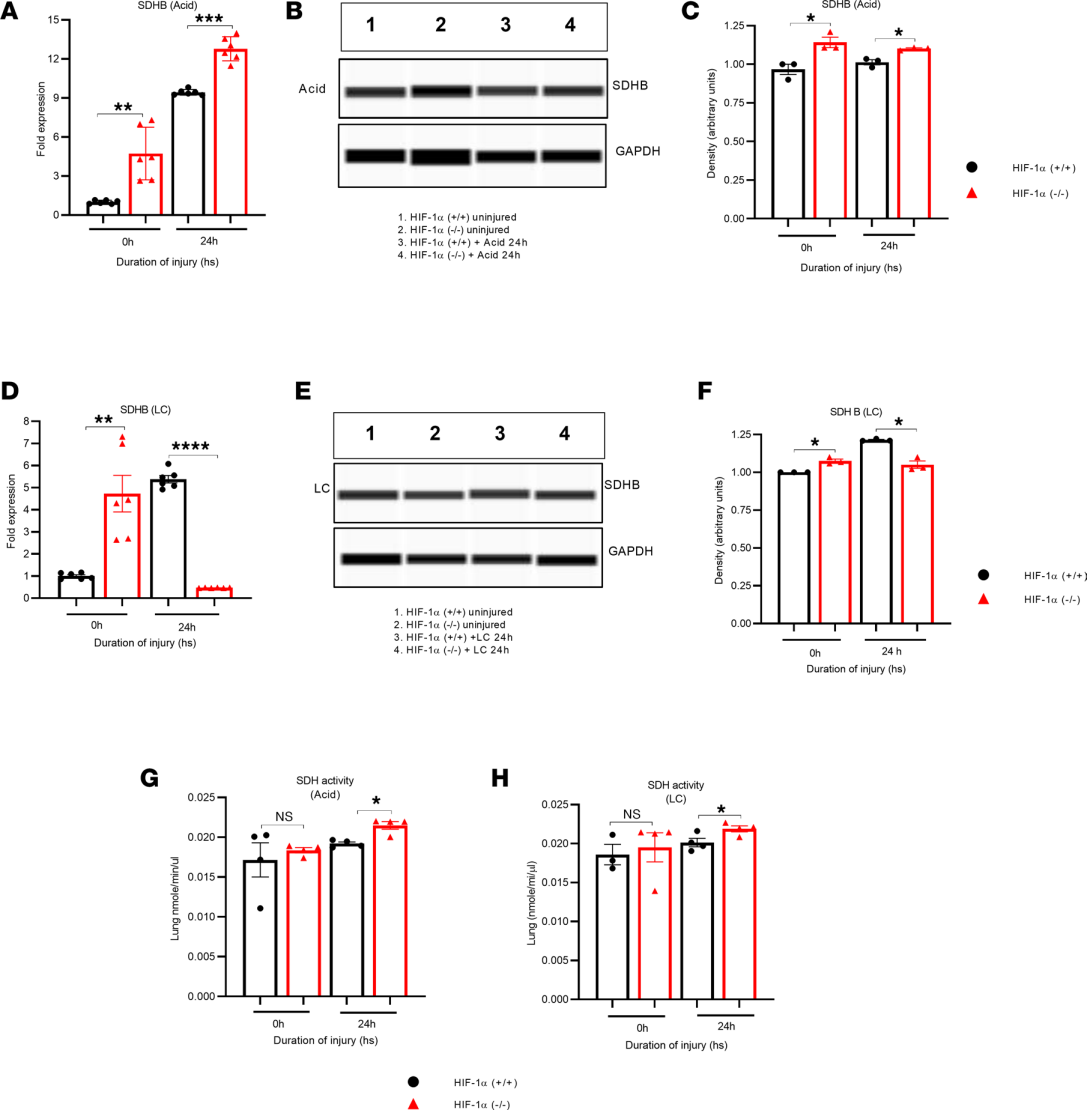
The absence of HIF-1α resulted in elevated SDH activity following LC and GA. HIF-1α^+/+^ and HIF-1α^–/–^ mice were subjected to LC or GA (*n* = 6). (**A**–**C**) We employed qPCR to identify the presence of the SDHB gene expression (**A**) and Western blots by protein level (**B** and **C**) following GA. (**D**–**F**) We also measured the SDHB gene (**D**) and the protein expression through capillary Western blots after LC (**E** and **F**). The data for GAPDH, as shown in Figure 10B and **B** after GA and Figure 10E and **E** after LC, are identical due to the use of a single Western blot and the same samples before and after treatment with GA/LC. (**G** and **H**) The level of SDH activity was tested in HIF-1α^+/+^ and HIF-1α^–/–^ mice (*n* = 3) that were exposed to acid aspiration (**G**) and LC (**H**). After analyzing the data, we used the unpaired *t* test with Welch’s correction to determine its statistical significance. *P < 0.05, **P < 0.01, ***P < 0.001, ****P < 0.0001.

We examined the expression SDHB following acid aspiration and LC. There was a substantial increase in the SDHB (gene and protein level) expression in HIF-1α^–/–^ mice following acid aspiration compared with the HIF-1α^+/+^ mice (Figure 11, A–C). However, PCR and Western blot data reveal a reduction in the gene and protein expression of SDHB at 24 hours in the HIF-1α^–/–^ mice following LC compared with the HIF-1α^+/+^ mice (Figure 11, D–F). These data suggest that the reason succinate accumulates and triggers HIF-1α activation in lung injuries (such as LC and aspiration) may be a decrease in SDHA activity not the SDHB sub-unit.

**Figure 12:**
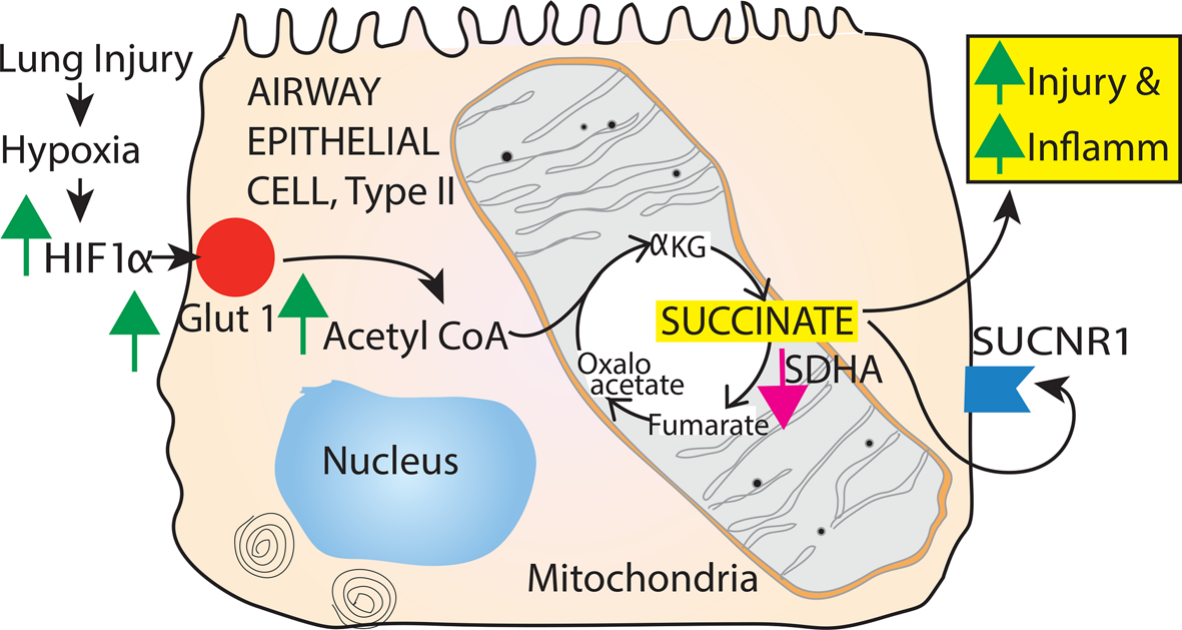
Type II AEC regulation of HIF-1α promotes lung injury and inflammation through succinate. This process is dependent on the activation of HIF-1α in Type II AEC. The process is intricately linked with the increased entry of glucose into the cell, mediated by GLUT-1. HIF-1α–regulated reduction in SDHA promotes extracellular succinate production. Succinate directly induced inflammation and injury to the surrounding AEC through the presence of SUCNR1 in AEC, thereby worsening permeability injury and inflammation.

Finally, we conducted a calorimetric assay to measure SDH activity in lung samples from 2 groups of mice (HIF-1α^+/+^ and HIF-1α^–/–^) after LC or GA. Our results indicate that, at 24 hours after LC or acid aspiration, the SDH activity was significantly greater in HIF-1α^–/–^ mice compared with the HIF-1α^+/+^ mice (Figure 11, G and H). Depicted in Figure 12 is a graphical overview of the role of succinate in driving HIF-1α–mediated lung injury and inflammation.

## Discussion

Blunt trauma–induced LC and GA are among the most common risk factors that initiate direct lung injury in a patient following vehicular motor accidents (3, 24). They are also one of the most prevalent risk factors for developing acute respiratory failure and ARDS. Hypoxia is these states’ most relevant physiologic dysfunction and is the primary determinant of mortality (25). Hypoxia resulting from these injuries demands respiratory assistance, including the need for mechanical ventilation, an intervention associated with considerable morbidity. Previous work in our lab demonstrated that HIF-1α increases in the lung and Type II AEC following LC and GA (9). Additionally, the downregulation of HIF-1α, specifically in AEC, reduced permeability and lung inflammation following direct unilateral (LC) and bilateral diffuse insult (GA) to the lung (9).

To investigate the effect of HIF-1α specifically in AEC, we sought to determine the contribution of glycolysis and mitochondrial oxidative phosphorylation to the worsening of inflammation and permeability injury. Metabolites associated with energy metabolism can affect every cellular function and integrity process. These metabolites have been extensively studied in cancer and immunologically active cells such as macrophages (26). Prolyl hydroxylase (PHD) activity is blocked by succinate and fumarate, stabilizing HIF-1α activity (23). Succinate, specifically in macrophages, stabilizes HIF-1α and is considered a predominant regulator of acute inflammation mediated by IL-1β (27). However, the effect of HIF-1α on succinate in the context of nonimmune cells, such as AEC, has not been fully explored. Specifically, we report that, in AEC, with the presence of HIF-1α, there are increased glycolytic and TCA cycle intermediates. The data support the notion that an increase in glycolytic and TCA intermediates due to enhanced glucose flux is substantially influenced by the presence of HIF-1α in Type II AEC. This finding differs from isolated cells subjected to hypoxia, where HIF-1α is considered adaptive and transforms the metabolism to predominant glycolysis. It is entirely possible that HIF-1α plays a role of an adaptive molecule by driving the metabolic pathway toward glycolysis in specific etiologies of lung injury (context-dependent role of HIF-1α). There is a body of evidence that shows that enhancement of glycolysis by inhibition of PHD, and thereby activation of HIF-1α, protects AEC from the adverse effects of lung injury (28, 29). Eltzschig and colleagues have reported that, in a model of alveolar stretch–induced inflammation, HIF-1α is protective (30). These results are contradictory to our experimental data. In experimental models employed in the current manuscript, hypoxia is a direct consequence of LC and acid aspiration. Patients with similar clinical conditions tend to deteriorate to require mechanical ventilation. Alveolar stretch is a phenomenon seen in ventilator-induced lung injury following the development of refractory hypoxia in lung injury. HIF-1α likely has disparate roles that depend on the cell type, nature of the inflammatory response in specific injuries, and timing of injury (acute versus chronic).

A factor that could lead to altered glycolysis and the TCA cycle involves the entry of glucose and its intracellular availability. Glucose is a primary source of metabolic energy for all mammalian cells and is primarily transported across the plasma membrane by facilitative glucose transporters (31). In the human airway epithelium (trachea, bronchi, and bronchioles), GLUT-1 is the predominant glucose transporter type expressed (32). Hypoxia is directly associated with increased mRNA of GLUT-1 in airway epithelial cells (33). A reduction in glucose transport into the cell can potentially reduce glucose metabolism in general. In conjunction with this prediction, we observed both transporter molecules (GLUT-1 and -4) involved in mammalian cells to be markedly reduced in the absence of HIF-1α.

Additionally, our results suggest that increasing molar concentrations of lactate in human airway epithelial cells in vitro did not directly activate IL-1β independently of hypoxia. These results are in concord with published data indicating the mitigation of inflammatory response to LPS in macrophages by lactate (34). These data suggest that lactate does not play a direct role in the initiation and progression of the inflammatory response in AEC.

We performed a similar experiment on human airway epithelial cells. We studied the effect of hypoxia and increasing concentrations of diethyl succinate (cell membrane is permeable) on the generation of HIF-1α and IL-1β. The combination of hypoxia and diethyl succinate profoundly increased the generation of both HIF-1α and IL-1β, and diethyl succinate directly increased the activation of both HIF-1α and IL-1β. The only TCA intermediate known to stabilize HIF-1α, fumarate, has profound antiinflammatory effects (17). In conjunction with in vivo data in triple transgenic HIF-1α mice, these data strongly indicate that succinate plays a direct role in the AEC regulation of inflammation and injury.

SUCNR1 is located in the plasma membrane and is a sensor for extracellular succinate (35). Over the last decade, it has become clear that SUCNR1 participates in multiple processes; is present in various tissues, including airways; and is a valid therapeutic target. Like most GPCRs, SUCNR1 is also subject to desensitization with prolonged signaling (35). A recent study indicates that increased plasma succinate in patients with critical injuries activates SUCNR1 and subsequent neutrophil activation (36). We observed considerable SUCNR1 protein expression in both whole lungs, isolated Type II AEC, and A549 cells.

Succinate metabolism is partly mediated by the enzyme SDH (22). SDH is a component of respiratory complex II found in bacterial cells and the inner mitochondrial membrane of eukaryotes (22). SDH is responsible for the oxidation of succinate to fumarate. Inhibition of SDH increases succinate accumulation. Following LC and acid aspiration (along with uninjured controls) in HIF-1α^+/+^ and HIF-1α^–/–^ mice, we analyzed whole-lung samples for genes and protein expression of SDHA and SDHB. Activation of HIF-1α in Type II AEC was associated with significantly lower gene and protein expression of SDHA in both LC and acid injury. While the expression of the SDHB subunit with acid injury was similarly dependent on HIF-1α, the results were the exact opposite in LC. These data indicate that the mechanism of succinate accumulation with activation of HIF-1α may be more linked to reduced SDHA activity in both acid-induced lung injury and LC. It is also evident that the mechanism of these injuries and the regulation of SDH may be different and contextually defined by the type of insult.

In conclusion, we report that hypoxia in mice following direct lung injury promotes the accumulation of succinate. This process is dependent on the activation of HIF-1α in Type II AEC. The process is facilitated by a reduction in SDHA that promotes extracellular succinate production. Succinate directly induced inflammation and injury to the surrounding AEC through the presence of SUCNR1 in AEC, thereby worsening inflammation and permeability injury (Figure 9). We believe the findings presented in the current manuscript are the first to describe the direct role of succinate in generating inflammation in nonimmune cells in direct lung injury.

## Methods

### HIF-1α mice.

Triple transgenic mice were created by mating HIF-1_flox/flox and SP-C-rtTA_/tg/(tetO)7-CMV-Cretg/tg transgenic mice. The generated mice, SP-C-rtTA_/tg/(tetO)7-CMV-Cretg/tg/HIF-1_flox/flox, are capable of respiratory epithelium-specific conditional recombination in the floxed HIF-1α gene upon exposure to doxycycline (a gift from John J. LaPres, Department of Biochemistry and Molecular Biology at Michigan State University [MSU], East Lansing, Michigan) (14, 23). The HIF-1_flox/flox mice were initially maintained in a C57BL/6 genetic background, while the SP-C-rtTA_/tg/(tetO)7-CMV-Cretg/tg mice was generated in an FVB/N genetic background. Aspiration was produced in Type II AEC–specific HIF-1α–cKO age-matched (6–8 weeks) triple transgenic mice (SP-C-rtTA_/tg/[tetO]7-CMV-Cretg/tg/HIF-1_flox/flox), which were previously used, and control mice (9, 12). Additionally, male and female age-matched (6–8 weeks, bred in-house) C57BL/6 (The Jackson Laboratory) mice were used.

### Doxycycline treatment to achieve recombination in HIF-1α mice.

Postnatal recombination was performed by exposing lactating dams to feed containing doxycycline (625 mg/kg, Harlan Teklad) and drinking water (0.8 mg/mL, MilliporeSigma). Triple transgenic mice were then maintained on the same doxycycline-containing food and water until they were 7 weeks of age. Doxycycline treatment was terminated 7–10 days before GA. Throughout the paper, these mice will be referred to as HIF-1α^–/–^. HIF-1α^+/+^ (experimental control) used in the study were triple transgenic (SP-C-rtTA_/tg/[tetO]7-CMV-Cretg/tg/HIF-1flox/flox) mice that were maintained on regular food and water ad libitum (9, 12).

### A murine model for GA.

The acid component of gastric aspirates is frequently modeled by intratracheal instillation of hydrochloric acid (HCl) in animals. Lung injury in mice following GA of dilute HCl (ACID, normal saline [NS] + HCl; final pH 1.25) is characterized by a biphasic response. C57BL/6, ODD-Luc, HIF-1α^+/+^, and HIF-1α^–/–^ mice, weighing 20–25 g (6–8 weeks old, bred in-house) were used for ACID induction. Mice were anesthetized with 5% isoflurane in oxygen at a rate of 5 L/min. After installation of anesthesia, mice were injected with 30 μL of either normal saline, pH 5.3 (NS, vehicle control), or NS+HCl, pH 1.25 (ACID), via deep oral injection into the trachea. Animals were allowed to recover spontaneously (9).

### Murine model for LC.

HIF-1α^+/+^ and HIF-1α^–/–^ (6–8 weeks, bred in-house) mice were anesthetized, and LC was induced. Briefly, after induction of anesthesia, each mouse was placed in a left lateral position and injured with a cortical contusion impactor. The right chest was struck along the posterior axillary line 1.3 cm above the costal margin with a velocity of 5.8 m/s adjusted to a depth of 10 mm. Mice are then allowed to recover spontaneously (37, 38).

### Administration of anesthetic, analgesic, and resuscitation.

Animals were anesthetized by i.p. injection of ketamine (80–120 mg/kg body weight) and xylazine (5–10 mg/kg body weight) or isoflurane inhalation. Systemic analgesics were not used because of their effects on the immune/inflammatory system. Animals were humanely euthanized in severe respiratory distress beyond 48 hours (39).

### Liquid chromatography–MS/MS.

After LC, Type II AEC were extracted from HIF-1α^+/+^ and HIF-1α^–/–^ samples. The AEC were washed with PBS and immediately frozen using liquid nitrogen. We isolated Type II cells from mice at different time points after LC and followed the prescribed sample preparation guidelines outlined in ref. 40. Cells were then scraped into a 0.5 mL mixture of 1:1 water/methanol and subjected to liquid chromatography–MS as described previously (41). Data were processed using Mass Hunter Quantitative Analysis, version B.07.00. Metabolites were normalized to the nearest isotope-labeled internal standard and quantitated using a linear calibration curve. Briefly, liquid chromatography–MS was performed on an Agilent system consisting of a 1260 ultra-performance liquid chromatography module coupled with a Agilent 6520 quadrupole TOF mass spectrometer. The Gly/TCA/Nuc analysis in cultured cells was done by liquid chromatography–MS, and the protein levels were normalized to pmol per μg of protein.

### Glucose flux studies.

To test the impact of glucose on serum-fasted Type II AEC, we added 5 mM ^13^C_6_-glucose (CLM 1396, Cambridge Isotope Laboratories) in DMEM, with no glucose (11966025, Thermo Fisher Scientific), for 60 minutes. Afterward, we removed the medium and washed the cells with 150 mM ammonium acetate in liquid chromatography–MS grade water. We then harvested the cells with 200 μL of ice-cold methanol and froze them at –80°C until sample preparation. To extract the samples, we added 200 μL of cold water to the cells and scraped them, followed by sonication on ice for 10 seconds. We then added 400 μL of chloroform to the homogenate and centrifuged it at 17,000*g* for 10 minutes at 4°C. The resulting top layer was collected and dried with nitrogen.

### Metabolomics analysis of glucose flux studies.

To analyze the metabolites, we reconstituted samples in 30 μL of 2:1 mixture of acetonitrile and water, filtered them, and injected 5 μL for analysis. The samples were separated according to the previously described method (42) and analyzed on the Agilent 6546 quadrupole TOF mass spectrometer, which was coupled to Agilent 1290 LC. The gas temperature was set at 225°C, the drying gas flow at 10 L/min, the nebulizer at 40 psi, the sheath gas temperature at 300°C, and the sheath gas flow at 12 L/min. The Fragmentor (Agilent) was set at 125 V, the skimmer at 65 V, and VCap at 3,000 V. For verification of metabolite identity and retention time, authentic standards of all measured metabolites were run separately and spiked into pooled samples. All experiments were performed in triplicate.

### Determination of cytokine levels in cell culture medium.

To determine the soluble concentrations of IL-1β in the cell culture medium, we utilized ELISA. As previously described (13), the antibodies and recombinant cytokines needed for these assays were obtained from R&D Systems.

### SDH activity assay.

Lung tissue were collected from HIF-1α^+/+^ and HIF-1α^–/–^ mice to perform the SDH activity assay, following manufacturer instructions (MAK 197; Sigma-Aldrich).

### Succinate colorimetric assay.

We isolated lung tissue from HIF-1α^+/+^ and HIF-1α^–/–^ mice to perform the succinate assay. We followed the instructions provided by the manufacturer (MAK 184; Sigma-Aldrich) throughout the process.

### Glucose measurement.

The glucose level in lung tissues were measured in HIF-1α^+/+^ and HIF-1α^–/–^ mice according to the manufacturer’s instructions (MAK181; Sigma-Aldrich).

### TaqMan quantitative PCR.

Total RNA was prepared from whole-lung lysates and reverse transcribed into cDNA using M-MLV reverse transcriptase (Invitrogen). The cDNA was amplified by real-time quantitative TaqMan PCR using an ABI. Prism 7700 (Applied Biosystems) sequence detection system. TaqMan gene expression reagents or SYBR Green Master PCR mix (Applied Biosystems) were used to detect the genes responsible for inflammation. GAPDH was analyzed as an internal control. Data were expressed as the fold change in transcript expression. The fold difference in mRNA expression between treatment groups was determined by software developed by Applied Biosystems (8).

### Capillary Western blot.

Capillary Western blot analysis was performed with a Wes system (004–600, Protein Simple) according to the manufacturer’s instructions using a 12–230 kDa separation module (SM-W004, Protein Simple) and an anti-mouse detection module (DM-002, Protein Simple). The samples, blocking reagent (antibody diluent), primary antibodies (1:50 dilution), HRP-conjugated secondary antibodies (1:10, anti–rabbit secondary antibody, 042-206, Protein Simple), and chemiluminescent substrate were pipetted onto the plate of the separation module (13, 43). The following primary antibodies were used: HIF-1α (1:50, NB100-105, Novus Biologicals), VEGF (1:50, sc-7269, Santa Cruz Biotechnology Inc.), FIH-1 (1:50, sc-271780, Santa Cruz Biotechnology Inc.), SUCNR1 (1:50, PA5-99450, Thermo Fisher Scientific), SDHA (1:50, 14865-1-AP, Thermo Fisher Scientific), SDHB (1:50, PA5-23079, Thermo Fisher Scientific), and SPC (1:50, sc-518029, Santa Cruz Biotechnology Inc.).

### Preparation and isolation of Type II AEC from mice.

Crude lung cell suspensions were prepared from male HIF 1α^+/+^ and HIF 1α^–/–^ following LC as previously described (9, 13). Briefly, mice were anesthetized and exsanguinated by opening the peritoneum and clipping the left renal artery, and the lungs were perfused with PBS. The lungs were filled with 1.5 mL dispase via the tracheal catheter and then allowed to collapse naturally. Then, the lungs were immediately covered with crushed ice and incubated for 2 minutes. The lungs were then removed and placed in a 2 mL dispase in a 12 mL polypropylene culture tube, incubated for 45 minutes at room temperature, and put on ice until the next step. The lungs were transferred to DMEM with 0.01% DNase I in a 100 mm Petri dish. The digested tissue was carefully collected from the airways with the curved edge of curved fine-tipped forceps and gently swirled for 5–10 minutes.

The suspension was successively filtered through 100 and 35 μm nylon filters and then 15 μm nylon mesh. The filtered cell suspension was centrifuged at 130*g* for 8 minutes at 4°C and resuspended in the culture media. The cells were incubated with biotinylated anti–mouse CD-32 (553143, 2.4g2, BD Pharmingen), and anti–mouse-CD45(553078, 30-F11, BD Pharmingen) antibodies for 30 minutes at 37°C. Meanwhile, streptavidin-coated magnetic particles were washed in a culture medium with a polypropylene culture tube using a magnetic tube separator. After incubation, the cells were centrifuged (130*g* for 8 minutes at 4°C), resuspended in 7 mL DMEM, added to the magnetic particles, and incubated with gentle rocking for 30 minutes at room temperature. The tube was attached to the magnetic tube separator with adhesive tape for 15 minutes following the incubation. The cell suspension was aspirated from the bottom of the tube using a narrow-stemmed transfer pipet, centrifuged (750*g* at 4°C for 10 minutes), and resuspended in culture media. Cultures were incubated in a humidified, 10% CO_2_ chamber at 37°C and observed daily by phase-contrast microscopy. The protocol utilizes floating cells (Type II AEC), whereas immunologically active cells like macrophages and fibroblasts adhere to the culture dish. Next, cells were washed to evaluate cell yield, viability, and purity and to measure surfactant secretion. The cultures were washed twice to collect the Type II cells for RNA isolation.

### Cell culture.

Normal HSAEC (FC-0016, Lifeline Cell Technology) and Human lung epithelial cells (A549) (ATCC) were grown as a monolayer in 5% CO_2_ at 37°C in FK12 medium supplemented with 10% heat-inactivated FBS, 50 U/mL penicillin, and 50 mg/mL streptomycin. Cells were plated in 6-well culture plates for various experiments (9, 43).

### Induction of hypoxia.

A hypoxic condition was created using a hypoxic incubator. To induce hypoxia, HSAEC and A549 cells were placed in a hypoxic chamber with an oxygen concentration maintained at 1% O_2_ and 37°C. Following hypoxia, the cells were collected at 6, 24, and 48 hours. The cell lysate was collected for Western blot, and RNA was collected for quantitative PCR (44).

### A549 cells and acid treatment.

A549 cells were seeded in 6-well plates. After 24-hour incubation, cells were treated with HCl (pH 4.0, DMEM) for 1 hour at 37°C in 5% CO_2_. Control cells were exposed to PBS. After incubation with HCl, the acidified medium was discarded, and the cells were washed 3 times with complete media to confirm neutralization. Cells were washed with PBS. The cell lysate was collected and used for capillary Western blot (13).

### Statistics.

Data are expressed as mean ± SEM. Statistical significance was estimated using a 1-way ANOVA with GraphPad Prism 8 software (GraphPad Software Inc.). We analyzed the statistical significance of the data using 2-way ANOVA with Tukey’s multiple-comparison tests. We also used the 2-tailed, unpaired *t* test with Welch’s correction for individual-group comparisons. Analyses were run at a significance level of *P* < 0.05.

### Study approval.

All procedures performed were approved by the IACUC at the University of Michigan and complied with the state, federal, and NIH regulations.

### Data availability.

Values for all data points found in graphs are in the Supporting Data Values file.

## Author contributions

Conception and design were contributed by MVS, SP, and KR. Performed research was contributed by MVS, SA, GY, SS, DTS, and MSA. Analysis and interpretation were contributed by MVS and KR. Drafting of the manuscript for intellectual content was contributed MVS, SA, SP, and KR.

## Supplementary Material

Supporting data values

## Version 1 09/22/2023

Electronic publication
